# Neural network analysis as a novel skin outcome in a trial of belumosudil in patients with systemic sclerosis

**DOI:** 10.1186/s13075-025-03508-9

**Published:** 2025-04-11

**Authors:** Ilayda Gunes, Elana J. Bernstein, Shawn E. Cowper, Gauri Panse, Niki Pradhan, Lucy Duran Camacho, Nicolas Page, Elizabeth Bundschuh, Alyssa Williams, Mary Carns, Kathleen Aren, Sarah Fantus, Elizabeth R. Volkmann, Heather Bukiri, Chase Correia, Vijaya B. Kolachalama, F. Perry Wilson, Seamus Mawe, J. Matthew Mahoney, Monique Hinchcliff

**Affiliations:** 1 Department of Internal Medicine, Section of Rheumatology, Allergy & Immunology, Yale School of Medicine, New Haven, USA; 2 Department of Medicine, Division of Rheumatology, Columbia University Irving Medical Center, New York, USA; 3 Departments of Dermatology and Pathology, Yale School of Medicine, New Haven, USA; 4 Department of Medicine, Division of Rheumatology, Northwestern University Feinberg School of Medicine, Chicago, USA; 5 David Geffen School of Medicine, Division of Rheumatology, University of California, los Angeles, Los Angeles, USA; 6 Boston University, Boston, USA; 7 Clinical and Translational Research Accelerator, Yale School of Medicine, New Haven, USA; 8 Department of Internal Medicine, Section of Nephrology, New Haven, USA; 9 The Jackson Laboratory, Bar Harbor, USA

**Keywords:** Systemic sclerosis, Scleroderma, Modified Rodnan skin score, Deep Neural Network, AlexNet, Belumosudil, Outcome measure, Outcomes, Artificial intelligence, Dermal fibrosis, Skin fibrosis

## Abstract

**Background:**

The modified Rodnan skin score (mRSS), a measure of systemic sclerosis (SSc) skin thickness, is agnostic to inflammation and vasculopathy. Previously, we demonstrated the potential of neural network-based digital pathology applied to SSc skin biopsies as a quantitative outcome. Here, we leverage deep learning and histologic analyses of clinical trial biopsies to decipher SSc skin features ‘seen’ by artificial intelligence (AI).

**Methods:**

Adults with diffuse cutaneous SSc ≤ 6 years were enrolled in an open-label trial of belumosudil [a Rho-associated coiled-coil containing protein kinase 2 (ROCK2) inhibitor]. Participants underwent serial mRSS and arm biopsies at week (W) 0, 24 and 52. Two blinded dermatopathologists scored stained sections (e.g., Masson’s trichrome, hematoxylin and eosin, CD3, α-smooth muscle actin) for 16 published SSc dermal pathological parameters. We applied our deep learning model to generate QIF signatures/biopsy and obtain ‘Fibrosis Scores’. Associations between Fibrosis Score and mRSS (Spearman correlation), and between Fibrosis Score and mRSS versus histologic parameters [odds ratios (OR)], were determined.

**Results:**

Only ten patients were enrolled due to early study termination, and of those, five had available biopsies due to fixation issues. Median, interquartile range (IQR) for mRSS change (0–52 W) for the ten participants was -2 (-9—7.5) and for the five with biopsies was -2.5 (-11—7.5). The correlation between Fibrosis Score and mRSS was R = 0.3; *p* = 0.674. Per 1-unit mRSS change (0–52 W), histologic parameters with the greatest associated changes were (OR, 95% CI, *p*-value): telangiectasia (2.01, [(1.31—3.07], 0.001), perivascular CD3 + (0.99, [0.97—1.02], 0.015), and % of CD8 + among CD3 + (0.95, [0.89—1.01], 0.031). Likewise, per 1-unit Fibrosis Score change, parameters with greatest changes were (OR, *p*-value): hyalinized collagen (1.1, [1.04 – 1.16], < 0.001), subcutaneous (SC) fat loss (1.47, [1.19—1.81], < 0.001), thickened intima (1.21, [1.06—1.38], 0.005), and eccrine entrapment (1.14, [1—1.31], 0.046).

**Conclusions:**

Belumosudil was associated with non-clinically meaningful mRSS improvement. The histologic features that significantly correlated with Fibrosis Score changes (*e.g.,* hyalinized collagen, SC fat loss) were distinct from those associated with mRSS changes (*e.g.,* telangiectasia and perivascular CD3 +). These data suggest that AI applied to SSc biopsies may be useful for quantifying pathologic features of SSc beyond skin thickness.

**Supplementary Information:**

The online version contains supplementary material available at 10.1186/s13075-025-03508-9.

## Background

Systemic sclerosis (SSc) is a rare chronic autoimmune disease whose pathogenesis involves fibrosis, inflammation, and vasculopathy including vascular pruning and intimal thickening [1]. Systemic sclerosis subsets [limited cutaneous and diffuse cutaneous (dc)] are defined using the modified Rodnan skin score (mRSS), a semi-quantitative assessment of dermal thickness [0 (normal) to 3 (hidebound) for 17 body sites (range of 0 −51)] [2]. The score initially included assessment of 26 body areas, but nine (neck, upper back, lower back, and bilateral toes, shoulders and breasts) were dropped due to high inter-rater variation [3]. The construct validity of the mRSS is supported by the strong correlation (r = 0.81) between forearm skin scores and dry weights of adjacent 7-mm punch biopsies [3]. The mRSS is currently the gold standard for assessing skin thickness in patients with SSc [4, 5]. However, despite promising pre-clinical data, results of clinical trials whose primary endpoint is the mRSS have been uniformly negative [6–21]. We wondered if negative trial results could be due, at least in part, to the mRSS being an incomplete readout of clinical changes as opposed to ineffective therapies.

Belumosudil, an inhibitor of Rho-associated coiled-coil containing protein kinase 2 (ROCK2), was approved by the US Food and Drug Administration in 2021 for chronic graft-versus-host-disease (cGVHD) treatment [22], a disease with similar histopathological features as SSc. Belumosudil downregulates proinflammatory responses by inhibiting signal transduction and activation of transcription 3 (STAT3) phosphorylation, upregulating STAT5 phosphorylation, and shifting T helper 17 (Th17)/T regulatory (Treg) balance towards the Treg phenotype: all mechanisms that should improve SSc skin disease [23].

Artificial intelligence technology has been successfully applied in many fields including biomedical research and medical education to name a few [24, 25]. For example, AI has been shown to aid diagnosis, reduce medical errors, and improve medical education [26, 27]. We previously subjected forearm skin biopsies to AI methods for skin disease measurement in patients with SSc [28]. Briefly, we applied a pre-trained deep neural network (DNN AlexNet) to 100 randomly selected dermal image patches (~ 0.16 mm^2^) per biopsy and generated 4,096 quantitative image features (QIFs) per image patch (409,600 QIF/biopsy). We used QIF signatures/biopsy to develop two regression models: 1) to predict whether a biopsy was from an SSc patient versus a healthy control (HC), and 2) to predict the mRSS (the output of this model is termed ‘Fibrosis Score’). The present research goal was to gain insights into the SSc histopathology features quantified by the DNN algorithm.

Here, we stained skin biopsies from belumosudil clinical trial participants, applied the AlexNet DNN algorithm, and generated Fibrosis Scores for each biopsy. Concurrently, we applied additional stains to skin sections, quantified SSc histopathological features using standardized approaches, and assessed the association between SSc histopathological features and the mRSSs and the Fibrosis Scores [5]. In a small number of patients treated with belumosudil, we found non-clinically meaningful mRSS improvement. Importantly, we found that the DNN algorithm applied to stained SSc skin section may be a reproducible, quantitative outcome for SSc skin disease in a clinical trial. Moreover, the histologic features that significantly correlated with mRSS changes (*e.g.,* telangiectasia and perivascular CD3 +) appear distinct from those that significantly correlated Fibrosis Score changes (*e.g.,* hyalinized collagen, SC fat loss). These results suggest that applying AI such as DNN algorithms to skin sections from patients with dcSSc may be a potentially useful quantitative outcome for measuring SSc skin disease beyond thickness.

## Methods

### Participants

The early phase 2, open-label, belumosudil multicenter study protocol (Kadmon Corporation LLC., a Sanofi company, KD025-215) received Institutional Review Board approval at each participating site (Yale University, Columbia University, Northwestern University, and University of California, Los Angeles). Patient research partners provided written informed consent in accordance with the Declaration of Helsinki. Patients with SSc who fulfilled the 2013 American College of Rheumatology/European League Against Rheumatism Systemic Sclerosis Classification Criteria [with SSc disease duration ≤ $6$ years and diffuse cutaneous disease (15 $\le$ mRSS $\le$ 40)] were eligible [5, 29] (Table 1).

**Table 1:** Baseline study participant data

**Characteristic, mean (SD) or as indicate**d		
	Trial Participants *N* = 10	Participants with paired skin biopsies *N* = 5
Age	49.6 (7.3)	53.2 (4.34)
dcSSc duration (y)	4.46 (1.01)	4.2 (0.84)
Female, n (%)	10 (100)	5 (100)
Race/Ethnicity, n (%)		
White, non-Hispanic	8 (80)	5 (100)
Black or African American, non-Hispanic	2 (20)	0 (0)
Positive Anti-Scl-70, n (%)	2 (25) n = 8	1 (20)
Positive Anti-RNA polymerase III, n (%)	4 (50) n = 8	3 (60)
Baseline FVC % predicted (L)	85 (20)	71 (25.9)
Baseline DLCO % predicted (L)	75 (14)	66.8 (7.8)
ILD on HRCT, n (%)	1 (11.1) n = 9	1 (20)
mRSS	24.9 (6.79)	24.4 (9.81)

*dcSSc* diffuse cutaneous systemic sclerosis, *Scl-70* anti-topoisomerase I antibodies, *FVC* forced vital capacity, *DLCO* diffusion capacity for carbon monoxide, *ILD* interstitial lung disease,*HRCT* chest high-resolution computed tomography, *mRSS* modified Rodnan Skin Score

### Clinical assessment and dermal biopsies

Consenting participants received 200 mg belumosudil tablets twice daily for 52 weeks. Clinical data including mRSS, and skin biopsies, were obtained. Participants underwent 4 mm dermal biopsies of the non-dominant dorsal mid-forearm at baseline (W0) and W24 and 52 and/or at end of trial (EOT). Biopsies were placed in formalin, transferred to 70% ethanol after 24 h, and shipped to a central laboratory for analysis. Biopsies were paraffin-embedded, sectioned and stained with hematoxylin and eosin (H&E), Masson’s trichrome, CD3, CD8, CD31, CD34, and alpha-smooth muscle actin (αSMA) (all Leica Biosystems, Buffalo Grove, IL). The AlexNet DNN algorithm was applied to trichrome-stained sections as previously described [28].

The primary trial outcome was the American College of Rheumatology (ACR)-endorsed Composite Response Index for Systemic Sclerosis (CRISS) response at W24 [30, 31]. Briefly, the CRISS is a two-step process with assessment of newly impaired or worsening cardiac function (ejection fraction of ⩽45% requiring treatment), lung function (relative loss of forced vital capacity % predicted (FVC) of > 15 in patients with ILD) or new onset of pulmonary arterial hypertension (PAH), or the occurrence of scleroderma renal crisis during step one. In the absence of step one outcomes, five variables [FVC% predicted, mRSS, Physician Global Assessment (PGA), Patient Global Assessment (PtGA) [32], and Health Assessment Questionnaire-Disability Index (HAQ-DI)] [33] are evaluated in step two, and the overall probability of improvement during the trial are reported. We anticipate future publication of the trial results.

### Histological analysis

**Fig. 1:**
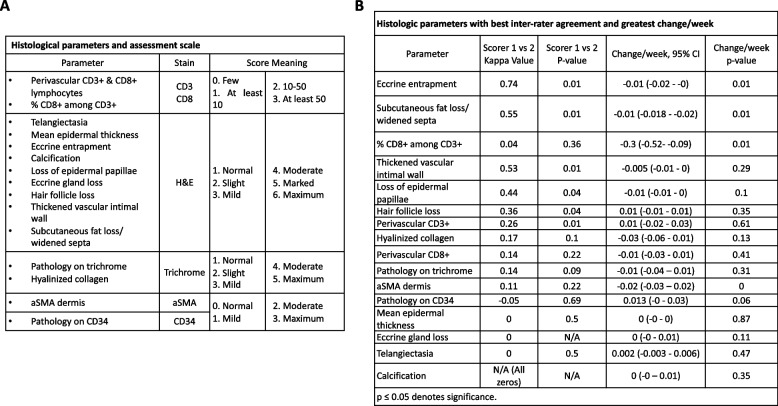
Systemic sclerosis dermatopathology parameters and scoring, inter-rater agreement and parameter change. **A** Scoring for 16 SSc dermatopathology parameters. **B** Histologic parameter Kappa values and parameter score change per week (*indicates *p*-value < 0.05)

Two blinded dermatopathologists independently scored slides for 16 histological parameters based upon previous SSc histopathology studies (Fig. 1a) [34–37]: 1) Epidermal papilla loss, 2) entrapment of at least one eccrine coil, 3) complete loss of eccrine coil, 4) presence of telangiectasia, 5) loss of at least one hair follicle, 6) presence of calcification, 7) loss of subcutaneous (SC) fat or widening of the SC septa, 8) presence of at least one thickened intima, all scored as “yes” or “no”; 9) mean epidermal thickness in µm (for five randomly selected sites); 10 & 11) density of perivascular CD3 + and CD8 + lymphocytes, 12) % CD8 + among CD3 + lymphocytes, 13) hyalinized collagen, 14) CD34, 15) trichrome, 16) αSMA staining (scored numerically with reference to a dermatopathologist-generated standard image set). Standard image set images were scored numerically from 0 = normal staining to 5 = maximum staining (Fig. 1a). To evaluate intra-observer reproducibility, each dermatopathologist reassessed histological features at two time points with a three-day washout period.

### Deep neural network feature extraction

**Fig. 2:**
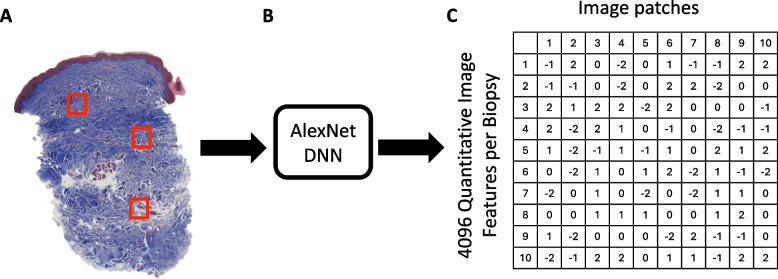
Deep Neural Network applied to trichrome-stained skin biopsy sections. **A** Image patches of skin biopsy section stained with trichrome. **B** Artificial intelligence (AlexNet Deep Neural Network) applied to each image patch (100 per biopsy). **C** Quantitative Image Features generated for each image patch

Skin biopsy sections were imaged using a Hamamatsu Nanozoomer S210 whole-slide scanner (Shizuoka, Japan) at 40 × objective, and images were saved as.ndpi files. Because the.ndpi file format is designed for storage, these images were converted into Zarr files for computational investigation. Zarr files were transformed into QIFs using the AlexNet DNN model, pretrained on ImageNet, available from the ONNX Model Zoo [38]. The AlexNet algorithm was applied to each of 100 randomly selected image patches (~ 0.16 mm^2^) from the dermis (epidermis and subcutis were excluded) to generate a new set of 4,096 QIFs for each image patch (100 × 4,096 QIF matrix) (Fig. 2).

### Linear regression model and association with mRSS and local skin score

As in prior work, we developed a linear regression model composed of QIF as predictor variables and the mRSS as the outcome variable (model output termed ‘Fibrosis Score’). We selected lambda from a grid of 16 values logarithmically spaced between 10^–5^ (weak penalty) and 10^3^ (strong penalty). For each image patch and value of lambda, the linear model predicted a value for mRSS as a linear combination of the QIF levels plus an intercept (which accounts for the mean mRSS of the training population). To integrate from image patches to biopsies, we averaged the predicted scores for the 100 image patches within a biopsy to obtain a Fibrosis Score. The model was cross validated by holding out sets of patients to avoid within-patient correlations.

### Statistical analyses

Inter- and intra-rater variability for 16 histological assessments were calculated using Cohen’s Kappa values [39]. We defined Kappa values ≤ 0 = no agreement, 0.01–0.20 = none to slight, 0.21–0.40 = fair, 0.41– 0.60 = moderate, 0.61–0.80 = substantial, and 0.81–1.00 = almost perfect agreement [39]. Ordinal logistic regression models were used to calculate odds ratios (OR), the relationship between an ordinal response variable (the mRSS and the Fibrosis Score, separately) and one or more explanatory variables (histological parameters). The dermatopathologists’ parameter score change/week average from their two timepoints was computed by fitting each measurement into a mixed effects model, with random effects for participant and slope and an independent covariance structure. Spearmen’s rank correlation coefficient was used to measure correlations between mRSS and the Fibrosis Score. As mentioned above, a lasso regression model trained using a previously validated data set to read trichrome-stained biopsy skin sections was applied to obtain a ‘Fibrosis Score’ (arbitrary range approximating the mRSS range 0–51) [28]. The Fibrosis Score was compared to mRSS through a correlation plot. A *p*-value < 0.05 was considered statistically significant. All statistical analyses were obtained using Stata, version 18 (Statacorp, College Station, TX). No corrections for multiple hypothesis testing were made due to the exploratory nature of this study.

## Results

### Participant data

**Fig. 3:**
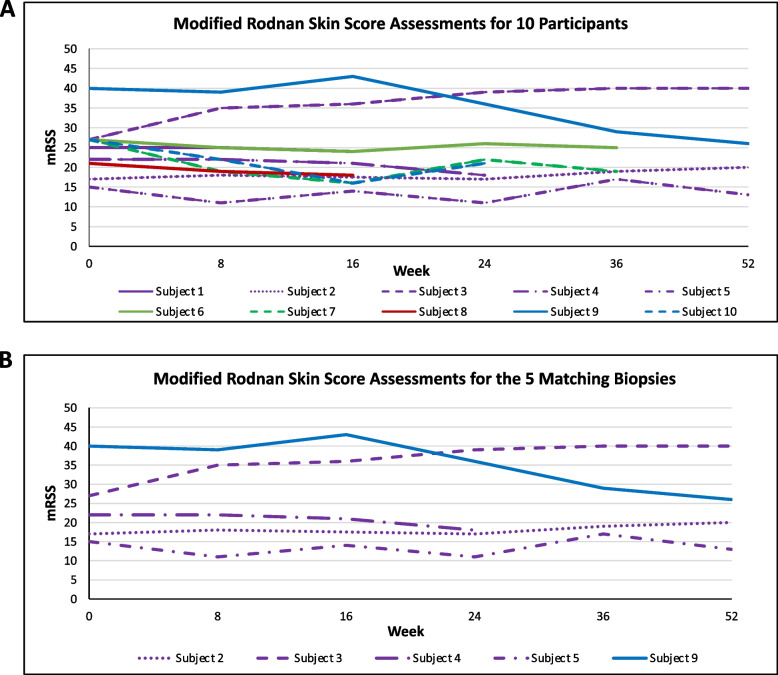
Modified Rodnan skin score trajectories. **A** Ten participants’ mRSS change from W0-52, median (interquartile range/IQR): −2.5 (−11—7.5). **B** Five participants’, with paired biopsies, mRSS change from W0-52, median (IQR): W0 to last follow-up: −2 (−9—7.5)

Ten participants were recruited for this pilot study designed to test preliminarily the safety of belumosudil for the treatment of skin fibrosis in patients with early dcSSc. The ten recruited patients were women, eight were white (non-Hispanic) and two were black (non- Hispanic) (Table 1). Participants had a mean (SD) age of 49.6 (7.3) years and dcSSc duration of 4.5 (1.0) years, respectively. The median, interquartile range (IQR) mRSS change from 0-52W was −2.5 (−11—7.5) (Fig. 3a).

**Fig. 4:**
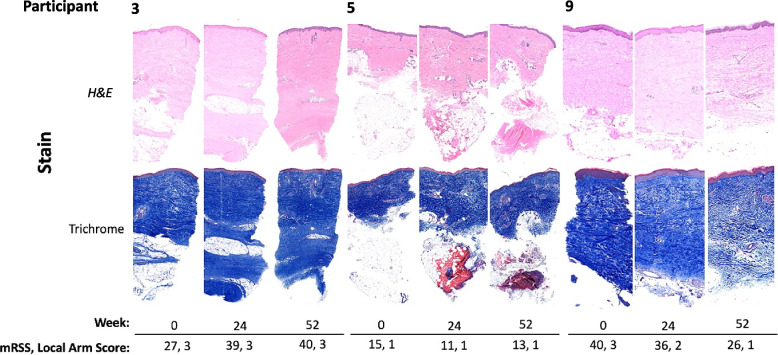
Stained sections for three participants. H&E and trichrome-stained biopsies for three participants with 0-, 24- and 52-W biopsies showing associated mRSS and local arm scores

Four out of ten participants completed the trial. Of the six participants who did not complete the trial: three withdrew due to adverse events, one had early study termination due to adverse events, one had early study termination due to progressive disease, and one patient died of acute renal failure and shock (deemed unrelated to the study drug). Skin biopsies were collected from seven of ten participants using biopsy kits provided by a contracted research organization. Despite this, tissue fixation issues for biopsies collected at each participating site resulted in only five of seven participants having at least one paired biopsy suitable for analysis. The median (IQR) mRSS change 0-52W for these five participants was −2 (−9—7.5) (Fig. 3b). Three of those five participants had skin biopsies from all three time points: W0, W24, and W52 (Fig. 4). The remaining two participants had available paired biopsies from W0 and W24, and W24 and W52, respectively.

Eight out of the ten trial participants had SSc-specific antibody data available. Of these, four had positive RNA polymerase III (four with negative tests), and two participants had positive Scl-70 (six with negative tests). Two participants lacked both RNA polymerase III and Scl-70 serum autoantibodies. We lacked antibody information for two patients from two different participating sites (Table 1). The five participants with paired biopsies had available SSc-specific antibody data: one had a positive Scl-70 (four with negative tests), and three participants had positive RNA polymerase III (two with negative tests).

### Histological analysis

Eccrine entrapment (substantial agreement), SC fat loss/widened septum (moderate agreement), thickened intima (moderate agreement), and loss of epidermal papillae (moderate agreement) were the histological parameters with best inter-rater agreement [39] (Fig. 1b). Out of the 16 SSc parameters, significant change per week (from baseline to W52) was observed for eccrine entrapment, SC fat loss/widened septum, and % CD 8 + among CD3 + lymphocytes (Fig. 1b).

### Relationship between mRSS and the Fibrosis Score

**Fig. 5:**
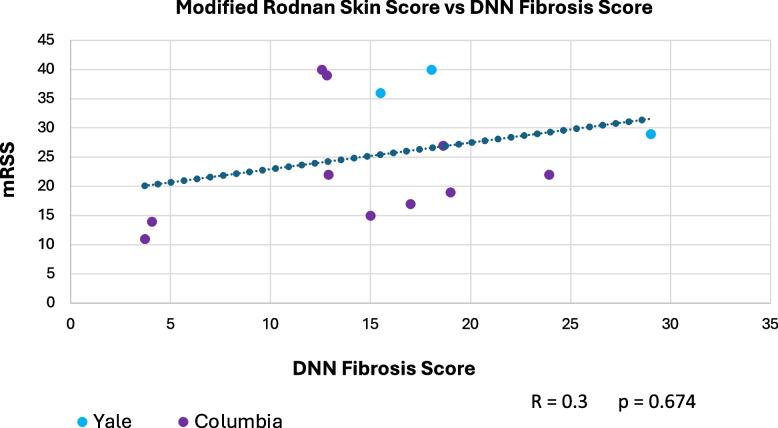
Correlation between modified Rodnan skin score (mRSS) and DNN Fibrosis Score. The Spearman correlation between mRSS and DNN Fibrosis Scores for five participants with baseline and follow-up biopsies W0-52

The correlation between the five participants’ mRSS (three time points each) and DNN generated Fibrosis Scores was R = 0.3 and *p* = 0.674 (Fig. 5).

### Relationship between mRSS and Fibrosis Score and histological parameters

Per 1-unit mRSS change, the histological parameters with significant associated responses/changes (OR, 95% CI, *p*-value) were: telangiectasia = 2.01, [1.31—3.07], (*p* = 0.001); perivascular CD3 + lymphocytes = 0.99, [0.97—1.02], (*p* = 0.015); and % of CD8 + among CD3 + cell = 0.95, [0.89—1.01], (*p* = 0.031). Similarly, per 1-unit Fibrosis Score change, the histological parameters with significant associated changes (OR, 95% CI, *p*-value) were: subcutaneous fat loss/widened septum = 1.47, [1.19—1.81], (*p* = 0.00033); thickened intima = 1.21, [1.06—1.38], (*p* = 0.005); eccrine entrapment = 1.14, [1—1.31], (*p* = 0.046), and hyalinized collagen = 1.1, [1.04 – 1.16] (*p* = 0.00033) (Table S1).

## Discussion

The mRSS, developed in the 1970s, remains the gold standard skin thickness assessment used in SSc clinical trials [3]. In spite of its inclusion as one of five components of the revised CRISS [4, 31], the mRSS has several limitations: it is only semi-quantitative, only assesses dermal thickness, requires long intervals between repeated measurements to observe clinically meaningful changes, and is confounded by obesity and edema [40]. Identification of a new SSc skin outcome that is quantitative, reproducible, sensitive to early changes, and inclusive of the three pathologic SSc features (fibrosis, inflammation and vasculopathy) would likely improve our ability to identify SSc skin disease. We used skin biopsies from a clinical trial to, 1) test the potential feasibility and utility of the DNN-derived Fibrosis Score as an SSc skin outcome, and 2) determine the histologic features (using published SSc histological features) that the DNN algorithm “sees” and quantifies. We found, 1) skin biopsies were feasible and useful to obtain during a clinical trial, 2) a weak correlation between the mRSS and the Fibrosis Score, and 3) different histological parameters were significantly associated with mRSS versus Fibrosis Score changes. These results suggest that skin biopsies could be included as an SSc clinical trial outcome.

Alternative approaches for quantifying SSc skin disease have been explored including durometry [41], optical coherence tomography [42], and histological readouts (hyalinized collagen, pathology on trichrome stain and loss of dermal papillae) [37]. Histological assessment of SSc skin was first described in a 1957 paper, where researchers showed the pathological features for a variety of clinical presentations of SSc to be indistinguishable [36]. Furthermore, the SSc microscopic features (*e.g.,* dermal edema, fibrosis, and sclerosis of collagen fibers, as well as obliterative changes of dermal vessels, elastic tissue loss, and loss of SC fat) varied depending on disease stage (*e.g.,* early edematous, sclerotic, or involutional) while epidermal changes were of limited diagnostic utility [36]. Results of a 2006 study suggested that myofibroblast number and hyalinized collagen alterations correlated with mRSS, durometric scores, and trichrome-stained skin biopsy scores [35], while results of a 2009 study suggested that myofibroblast number, narrowing of the arteriolar lumen in the deep vascular plexus (reticular dermis) and decreased dermal vascular density were significantly associated with increased skin thickness [34]. Results of a 2011 study reported that epidermal thinning, increased epidermal pigmentation, loss of epidermal papillae, and increased melanophages ('pigment incontinence') are important in SSc [37]. Based upon these four studies, our two study dermatopathologists selected 16 SSc skin disease histologic parameters (see Methods) to score. Parakeratosis, pigment incontinence, and mean epidermal pigmentation were excluded as they are confounded by native skin color and degree of external manipulation. Histologic parameters with best inter-rater consistency were eccrine entrapment, SC fat loss/widened septum, thickened intima, and loss of epidermal papillae. Histologic parameters with worst inter-rater consistency were pathology on CD34 staining, mean epidermal thickness, eccrine gland loss, and telangiectasia. Our results are consistent with histology results in other diseases where agreement among experts can be variable underscoring the need for assistive devices like AI [43].

We are the first to apply AI to SSc skin biopsies; however, AI methods are increasingly being applied to solve problems in medicine. For instance, investigators applied a pre-trained convolutional neural network model (trained on ImageNet) to H&E slides from tumors (*e.g.,* melanoma, lung) to determine the likelihood of response to anti-PD-1 treatment in patients. The model was used to classify each slide as belonging to an anti-PD-1 responder or a non-responder as assessed by progression-free survival. They calculated the area under the curve (AUC) comparing the model’s predictions to real-world outcomes. Results showed an AUC of 0.778 (95% confidence interval, 64%–91%) for 54 melanoma test samples and AUC of 0.645 (95% CI: 49% − 78%) for 55 lung cancer validation samples [44]. Another 2020 paper examined the performance of AI for prostate cancer detection compared to expert review by 23 urological pathologists. The AI system was trained using 6953 prostate biopsy cores stratified according to the Gleason score, a scoring system rating risk of cancer spread. The model was tested on an independent dataset comprised of 910 benign, and 721 malignant, biopsy slides and validated on an external dataset comprised of 108 benign, and 222 malignant, biopsy slides. The results showed an AUC representing the ability of the AI system to distinguish malignant from benign cores of 0.997 (95% CI 99.4%–99.9%) for the independent test dataset and AUC 0.986 (97.2%–99.6%) for the external validation dataset [43].

Applying AI (such as our DNN model) to skin biopsies as a novel SSc skin outcome would potentially improve clinical care and clinical trial design. Skin biopsies can readily be performed at medical centers around the globe, fixed in formalin, and shipped at room temperature to a central analysis site. This would drastically increase the number of recruitment sites and promote greater clinical trial participation by patients from diverse backgrounds for increased generalizability of trial data. Barring fixation issues such as we encountered, applying AI to skin biopsies from clinical trials should be feasible. Another benefit is the ability to train separate algorithms to quantify inflammation, vasculopathy, and/or increased dermal thickness depending on the mechanism of action of the therapy. How best to quantify the absence of a healthy skin histology feature (*e.g.,* loss of eccrine glands or hair follicles) will likely be more challenging.

We analyzed forearm skin biopsies because we, and others, have shown that forearm skin score (0–3) strongly and significantly correlates with mRSS [28, 45]. Thus, a quantitative AI-generated score of forearm skin disease could be a surrogate for total body skin disease. We view the low correlation between mRSS and Fibrosis Scores as a potential study strength because it suggests that our AI-generated score quantifies skin features beyond skin thickness. A strong correlation between the mRSS and Fibrosis Scores might suggest that the Fibrosis Score is merely a more precise quantification of skin thickening. Subcutaneous fat loss/widened septum, thickened intima, eccrine entrapment, and hyalinized collagen showed the strongest associations with the Fibrosis Score suggesting these parameters are important features in early dcSSc. Our small sample size precludes drawing firm conclusions, but our results provide an important proof-of-concept: clinical trials can include AI-generated skin outcome assessments that complement the mRSS. Of course, with any AI assessment of stained skin biopsies, staining batch effects must be addressed and overcome. This can be accomplished with larger sample sizes and inclusion of additional stains.

Eight of the ten participants demonstrated mRSS improvement between baseline and last assessment, but only four patients demonstrated clinically significant changes (defined as at least 20% or 25% improvement from baseline mRSS [30, 31]). Of note, each of these patients had RNA polymerase III serum antibodies present drawing into question whether the skin changes were due to treatment or due to the natural disease history. Two of these four patients showed mRSS reductions greater than five points, the minimal clinically important difference, thus supporting consideration of a larger, randomized, placebo-controlled trial of belumosudil.

We compared the histologic features that were significantly associated with the Fibrosis Score with those that were significantly associated with mRSS to assess concordance. Importantly, we found that mRSS improvement mirrored decreases in telangiectasia, perivascular CD3 + , and % CD8 + among CD3 + cells. Specifically, telangiectasia showed the highest odds of relative change compared to a 1-unit mRSS change [OR (95% CI) 2.01 (1.31 – 3.07)] (Table S1). Thus, each point increase in the mRSS increased the odds of having a higher telangiectasia score by 101%. Fibrosis Score changes were associated with hyalinized collagen, SC fat loss, thickened intima, and eccrine entrapment changes during belumosudil treatment. Specifically, SC fat loss/widened septum showed the highest odds of relative change compared to a 1-unit change in the Fibrosis Score [OR (95% CI) 1.47 (1.19—1.81)] (Table S1). Thus, each point increase in Fibrosis Score increased the odds of having a higher SC fat loss/widened septum score by 47%. These findings indicate that the mRSS and Fibrosis Score appear to measure distinct pathological features, and that combining the two approaches may be better than using either one in isolation. Thus, in addition to patient-reported outcome measures that assess treatment-associated feel and function changes, the best use of the Fibrosis Score presently would be inclusion as a complementary outcome to the mRSS.

Study limitations include the small dataset of only five participants with longitudinal biopsies. We planned to recruit twelve participants, but the study sponsor terminated the study after recruitment of ten. As stated above, three patients withdrew from the study and one patient died. Additionally, tissue fixation issues that we attribute to faulty skin biopsy collection kits resulted in 50% unusable biopsies (≥ 1 collected at each of the four participating sites). Small sample bias may have led to an overestimation of the odds ratios; therefore, larger studies are needed to validate these findings.

Study strengths include our multicenter study design, our application of a published AI approach for quantifying skin disease, and the use of real-world clinical trial biopsies. The comprehensive histologic assessments for 16 skin features by two dermatopathologists at two time points is an additional strength. We acknowledge that our previously published DNN model, utilized in this study, is based on a pre-trained neural network originally trained on natural images. Future iterations of our modeling framework will aim to incorporate more advanced techniques to fully capture the information within histological images. This may include developing more sophisticated frameworks such as graph neural networks [46–48] and improving averaging methods across selected patches to better represent the comprehensive details of the histological images.

With the growing number of proposed therapies for SSc skin disease, there is an urgent need to increase the number of investigative sites that can participate in trials. Our data demonstrate the feasibility of obtaining skin biopsies for use as SSc skin outcomes in clinical trials. Our research supports the combined use of DNN and histological parameter analyses of skin biopsies as feasible SSc skin outcomes that are quantitative and comprehensive for use in SSc clinical trials. Currently, we are analyzing archived biopsies from SSc patients obtained at other institutions, working to determine the optimal stain(s) (*e.g.,* CD3, CD8 and CD34) for use with AI, and annotating hundreds of slides for dozens of SSc features to permit us to train additional AI models with enhanced performance.

## Conclusions

To gain insights into the histological features of SSc that may be quantified using deep learning approaches, we applied our previously published DNN algorithm to stained skin biopsies obtained during a clinical trial of belumosudil in patients with early dcSSc. Our data show that belumosudil was associated with non-clinically meaningful mRSS improvement. We examined the relationship between the mRSSs and DNN-generated Fibrosis Scores, and important SSc histological parameters. The distinction between histologic parameters significantly associated with mRSS and Fibrosis Score suggests that the DNN algorithm may be a useful strategy for quantifying SSc pathologic features involved in SSc skin disease beyond from dermal fibrosis. Ongoing work includes analyses of larger cohorts and training the DNN algorithm with H&E-, in addition to trichrome-, stained samples. We herein present preliminary findings that support applying AI to stained skin biopsies in future SSc studies. This approach could potentially streamline clinical trials, transform the pace of global recruitment, and increase diversity and thus generalizability of SSc clinical trial results.

## Supplementary Information

Supplementary Material 1

## Data Availability

No datasets were generated or analysed during the current study.

## Abbreviations

SSc: Systemic sclerosis
lcSSc: Limited cutaneous systemic sclerosis
dcSSc: Diffuse cutaneous systemic sclerosis
mRSS: Modified Rodnan skin score
DNN: Deep neural network
QIFs: Quantitative image features
W: Week
EOT: End of trial
SC: Subcutaneous
AI: Artificial intelligence
ACR: American College of Rheumatology
CRISS: Composite Response Index in Systemic Sclerosis
FVC: Forced vital capacity
ILD: Interstitial lung disease
PAH: Pulmonary arterial hypertension
H&E: Hematoxylin and eosin
αSMA: α-Smooth muscle actin
IQR: Interquartile range
PGA: Physician global assessment
PtGA: Patient global assessment
cGVHD: Chronic graft-versus-host disease
ROCK2: Rho-associated coiled-coil containing protein kinase 2
STAT: Signal transducer and activator of transcription
