# Supplementary material for: Neural network analysis as a novel skin outcome in a trial of belumosudil in patients with systemic sclerosis
Source: Arthritis Res Ther. 2025 Apr 11;27:85. doi: 10.1186/s13075-025-03508-9 (PMC11987334; doi:10.1186/s13075-025-03508-9)
Supplement: Supplementary file 1 — Supplementary Material 1 [file 13075_2025_3508_MOESM1_ESM.pdf]

Supplementary Table 1

Multivariate Ordinal logistic Regression Model of the Relationship between mRSS and Fibrosis Score and Histological Parameters			
**Parameter**	**mRSS Odds Ratio (95% CI)**	**Parameter**	**Fibrosis Score Odds Ratio (95% CI)**
**Telangiectasia***	2.01 (1.31 - 3.07)	**Subcutaneous fat loss/widened septum***	1.47 (1.19 - 1.81)
**Perivascular CD3+ lymphocytes***	0.99 (0.97 - 1.02)	**Thickened intima***	1.21 (1.06 - 1.38)
**% of CD8+ among CD3+ cell***	0.95 (0.89 - 1.01)	**Eccrine entrapment***	1.14 (1 - 1.31)
**Eccrine entrapment**	1.2 (0.98 - 1.47)	**Hyalinized collagen***	1.1 (1.04 – 1.16)
**αSMA dermis**	1.26 (1.17 – 1.36)	**% of CD8+ among CD3+ cell**	1.13 (0.99 - 1.3)
**Eccrine gland loss**	1.21 (0.92 - 1.59)	**αSMA dermis**	1.37 (1.06 – 1.78)
**Thickened intima**	1.14 (1.03 - 1.27)	**Pathology on trichrome**	1.09 (1.01 - 1.18)
**Mean epidermal thickness**	1.07 (0.96 - 1.19)	**Telangiectasia**	1.01 (0.87 - 1.16)
**Pathology on trichrome**	1.06 (0.95 - 1.19)	**Perivascular CD8+ lymphocytes**	1 (0.81 - 1.23)
**Hyalinized collagen**	1.04 (0.93 - 1.17)	**Mean epidermal thickness**	0.94 (0.82 - 1.08)
**Perivascular CD8+ lymphocytes**	1.03 (1 - 1.06)	**Loss of epidermal papillae**	0.93 (0.72 - 1.21)
**Hair follicle loss**	1 (0.88 - 1.13)	**Eccrine gland loss**	0.91 (0.69 - 1.21)
**Subcutaneous fat loss/widened septum**	1.09 (0.93 - 1.28)	**Pathology on CD34**	0.9 (0.78 - 1.03)
**Pathology on CD34**	0.92 (0.83 - 1.01)	**Hair follicle loss**	0.89 (0.73 - 1.07)
**Loss of epidermal papillae**	0.9 (0.75 - 1.09)	**Perivascular CD3+ lymphocytes**	0.87 (0.68 - 1.11)
**Calcification**	N/A – All values are 0	**Calcification**	N/A – All values are 0

Histologic parameters (telangiectasia, perivascular CD3+ lymphocytes, and % CD8+ among CD3+ cells) with changes that were significantly associated with mRSS changes. Histological parameters (subcutaneous fat loss, thickened intima, and eccrine entrapment) with changes that were significantly associated with Fibrosis Score changes (Baseline, week 0, to last follow up) * denotes $p \leq 0.05$.
